# Natural variation in life history and aging phenotypes is associated with mitochondrial DNA deletion frequency in *Caenorhabditis briggsae*

**DOI:** 10.1186/1471-2148-11-11

**Published:** 2011-01-12

**Authors:** Suzanne Estes, Anna L Coleman-Hulbert, Kiley A Hicks, Gene de Haan, Sarah R Martha, Jeremiah B Knapp, Samson W Smith, Kevin C Stein, Dee R Denver

**Affiliations:** 1 Department of Biology, Portland State University, Portland, OR 97201, USA; 2 Department of Zoology and Center for Genome Research and Biocomputing, Oregon State University, Corvallis, OR 97331, USA

## Abstract

**Background:**

Mutations that impair mitochondrial functioning are associated with a variety of metabolic and age-related disorders. A barrier to rigorous tests of the role of mitochondrial dysfunction in aging processes has been the lack of model systems with relevant, naturally occurring mitochondrial genetic variation. Toward the goal of developing such a model system, we studied natural variation in life history, metabolic, and aging phenotypes as it relates to levels of a naturally-occurring heteroplasmic mitochondrial *ND5 *deletion recently discovered to segregate among wild populations of the soil nematode, *Caenorhabditis briggsae*. The normal product of *ND5 *is a central component of the mitochondrial electron transport chain and integral to cellular energy metabolism.

**Results:**

We quantified significant variation among *C. briggsae *isolates for all phenotypes measured, only some of which was statistically associated with isolate-specific *ND5 *deletion frequency. We found that fecundity-related traits and pharyngeal pumping rate were strongly inversely related to *ND5 *deletion level and that *C. briggsae *isolates with high *ND5 *deletion levels experienced a tradeoff between early fecundity and lifespan. Conversely, oxidative stress resistance was only weakly associated with *ND5 *deletion level while ATP content was unrelated to deletion level. Finally, mean levels of reactive oxygen species measured *in vivo *showed a significant non-linear relationship with *ND5 *deletion level, a pattern that may be driven by among-isolate variation in antioxidant or other compensatory mechanisms.

**Conclusions:**

Our findings suggest that the *ND5 *deletion may adversely affect fitness and mitochondrial functioning while promoting aging in natural populations, and help to further establish this species as a useful model for explicit tests of hypotheses in aging biology and mitochondrial genetics.

## Background

*Caenorhabditis elegans *has long been appreciated as a useful model organism for developmental, molecular, and aging biology. As molecular genetic and genomic approaches have become increasingly available, nematode biologists have also become interested in explaining and predicting natural patterns of phenotypic and genomic evolution. These interests have motivated recent efforts to quantify natural phenotypic variation and molecular population genetic structuring, and to characterize the ecology and natural history of nematode species in order to interpret such findings within robust population and ecological genetic contexts [e.g., [1-5]]. *Caenorhabditis briggsae *was until recently the closest known relative of *C. elegans *and has therefore been the subject of many different types of comparative analyses [e.g., [6-9]]. Building upon previous discoveries of mitochondrial DNA (mtDNA) variation segregating among natural populations [10,11], we sought to further develop the *C. briggsae *system by quantifying the degree to which this mtDNA diversity is associated with population-level variation in life-history, physiological, and aging related phenotypes. This work will provide a foundation for generating and testing hypotheses in mitochondrial mutation biology and aging research.

Mutations that impair functioning of the mitochondrial electron transport chain (ETC) are associated with a variety of human metabolic and age-related disorders [12-14]. A barrier to in-depth studies of the inheritance, population dynamics, and effects of clinically relevant mtDNA mutations has been the lack of genetic model systems that adequately represent the features of pathogenic mtDNA mutations [14]. Howe and Denver [10] discovered a naturally occurring heteroplasmic mtDNA deletion segregating among geographically distinct populations of the soil nematode, *Caenorhabditis briggsae*. The deletion eliminates the 5' end (first 786 bp) of the *NADH dehydrogenase subunit 5 *(*ND5*) gene, the normal protein product of which is an essential and highly conserved core subunit of mitochondrial ETC complex I [10]. In humans, several neurodegenerative disorders including Parkinson's disease are associated with heteroplasmic *ND5 *mutations [13].

**Figure 1:**
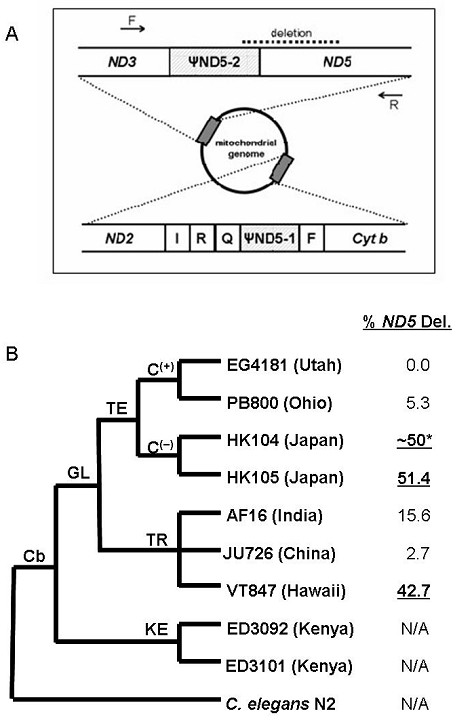
***C. briggsae *mitochondrial *ND5 *deletion**. A. *C. briggsae *mitochondrial genome positions of *ND5 *deletion (dashed line at top) and *ΨND5 *elements-pseudogenes that originated from duplications of *ND5*. Arrows = primers for PCR assays [reproduced from [10]]. B. *C. briggsae *intraspecific phylogeny with *C. elegans *as outgroup. GL = global intraspecific superclade; KE = Kenya clade; TE and TR = temperate and tropical subclades of GL. C(+) = temperate-clade isolates bearing DRSeq2 compensatory *ΨND5-2 *alleles; C(-) = those bearing ancestral alleles. % *ND5 *Del. indicates isolate-specific percentages of total mitochondrial genomes that harbor *ND5 *deletions as previously determined by qPCR [10] and confirmed by conventional PCR and gel analysis here (Additional file 1, Table S1). Isolates classified for statistical analyses as experiencing high (bold underlined font), low (normal font), or zero-*ND5 *("N/A") deletion levels are indicated on the phylogeny. *ND5 deletion levels for the HK104 isolate used here was estimated based on the standard PCR band size assay described in Howe and Denver [10] where the results of the standard PCR assay were shown to correlate positively and significantly with a qPCR method used to estimate deletion levels in the other isolates.

The *C. briggsae *deletion occurs as a consequence of directly repeated 21bp DNA sequence tracts in the *ND5 *gene and in an upstream pseudogene named *ΨND5-2 *(Figure 1A). The observed deletion is expected to strongly and negatively affect *ND5 *protein-coding function as the deleted sequences encode more than 200 ND5 amino acids, 34 of which are conserved in *C. elegans*, *D. melanogaster*, and humans. Two PCR approaches showed that deletion-containing genomes constitute from 0 to ~50% of total within-isolate mitochondrial populations (Figure 1B, Additional file 1, Table S1).

Based on other studies of ETC complex I-deficient genotypes [15-18], we hypothesized that expression of truncated *ND5 *protein products would be associated with mitochondrial dysfunction, increased reactive oxygen species (ROS) production, reduced fitness, and faster rates of aging. Higher rates of nuclear [6,19] and mitochondrial [20] mutation in *C. briggsae *relative to *C. elegans *and a negative correlation between *ND5 *deletion frequency and reproductive output in *C. briggsae *[10] are consistent with this hypothesis, although it cannot yet be ruled out that nuclear-encoded genes are responsible instead.

With the dual goals of further characterizing global phenotypic variation in *C. briggsae *and developing a novel model system for mitochondrial mutation dynamics, ETC dysfunction, and aging research, we performed a survey of natural variation in fitness, physiological, and aging related phenotypes among *C. briggsae *isolates containing different proportions of *ND5*-deletion bearing mtDNA genomes. We found significant among-isolate variation in all phenotypes studied, some of which was statistically associated with isolate-specific *ND5 *deletion heteroplasmy level. Specifically, *C. briggsae *isolates containing a high proportion of *ND5 *deletion genomes tended to have reduced reproductive fitness and early-onset physical decline compared to isolates with low or zero deletion levels. However, the same isolates did not differ consistently with respect to lifespan, ATP content, or acute oxidative stress resistance. Additionally, although the *C. briggsae *isolate with the highest *ND5 *deletion frequency exhibited the highest levels of ROS as expected, we found a non-linear relationship between *ND5 *deletion and ROS level. These patterns may be explained by as yet unknown differences in physiological adaptations among *C. briggsae *isolates; *e.g.*, ROS detoxification mechanisms and reliance on anaerobic energy metabolism.

## Results and Discussion

**Figure 2:**
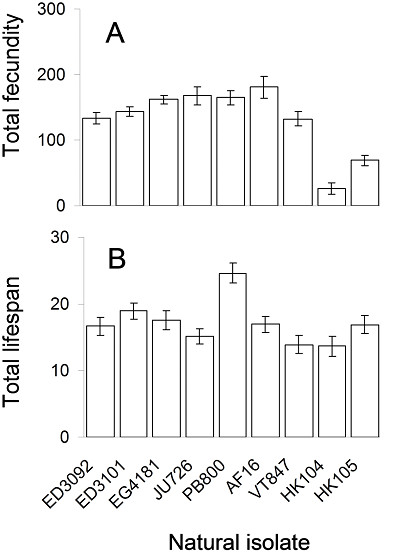
**Natural variation in life history traits**. Natural variation in mean lifetime fecundity (A) and mean lifespan (B) among *C. briggsae *isolates. Isolates are arranged along the x-axis in order of increasing *ND5 *deletion level. Bars show one standard error.

We surveyed variation in life history, physiology, and aging phenotypes among wild *C. briggsae *isolates that represent the full spectrum of natural variation in *ND5 *deletion frequency (Figure 1B). We found strong and significant negative correlations between *ND5 *deletion level and both nematode fecundity and population growth rate (*r*) (Table 1). These patterns are driven entirely by two of the three high-deletion isolates as compared to low and zero-deletion isolates (Figure 2A) and may indicate that the *ND5 *deletion has a negative effect on *C. briggsae *fitness only when its frequency reaches a certain threshold [cf. [21]]. Specifically, when isolates were subdivided into deletion frequency categories (Figure 1B legend), we found that high-*ND5 *deletion isolates had significantly reduced total fecundities and *r *compared to either low or zero-deletion isolates (Tukey HSD, α = 0.05; Figure 2A). We also found a significant negative correlation between *ND5 *deletion level and lifespan (Table 1, Figure 2B), but the pattern is far weaker than that for fecundity. Indeed, low-deletion isolates were found to live longer than either high or zero-deletion isolates (Tukey HSD, α = 0.05), a pattern driven by the particularly long lifespan of low-deletion isolate, PB800 (Ohio) (Figure 2B).

**Table 1:** Phenotypic variation among *C. briggsae *isolates

Character	Grand mean	SD	N	df	F	ρ
Early fecundity	31.99 surviving offspring	20.86	210	8	13.26***	-0.432**
Late fecundity	103.8 surviving offspring	58.03	210	8	14.93***	-0.482**
Total fecundity	135.7 surviving offspring	66.74	210	8	22.89***	-0.554***
Intrinsic rate of increase (*r*)	0.808	0.250	210	8	17.93***	-0.426***
Total lifespan	16.96 days	6.358	160	8	5.192***	-0.228***
Pharyngeal pumping	181.6 pumps/min	79.99	110	8	8.215***	-0.582***
ATP content	4.499 nM/mg protein	1.545	85	8	25.09***	-0.120
Paraquat resistance	61.03 min	21.70	127	8	7.139***	-0.204***
Superoxide level	224.20 relative fluorescence units	18.55	99	8	8.388***	0.119

Grand means, standard deviations, and sample sizes for all traits measured. F is the test statistic for one-way analyses of variance for each phenotype in *C. briggsae *isolates. ρ is the Pearson correlation coefficient between isolate-specific trait means and deletion frequencies. *, **, and *** denote significance at the 0.05, 0.01, and 0.001 levels, respectively.

**Figure 3:**
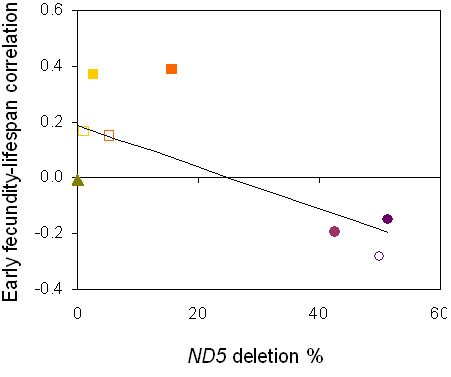
**Relationship between life-history tradeoffs and *ND5 *deletion frequency**. Linear regression of Spearman's correlation coefficients (ρ) between early fecundity and total lifespan for each *C. briggsae *isolate on isolate-specific *ND5 *deletion level. Slope (SE) = -0.0074 (0.0029), p = 0.036. Isolates are grouped as follows: zero deletion isolates ED3092 = open green triangle and ED3101 = filled green triangle (overlaps ED3092); low deletion isolates EG4181 = gold open square, JU726 = gold filled square, PB800 = orange open square, AF16 = orange filled square; and high deletion isolates VT847 = lavender filled circle, HK104 = purple open circle and HK105 = purple filled circle.

Consistent with findings from *C. elegans *[*e.g.*, [22,23]], we found little evidence for life history tradeoffs in *C. briggsae *isolates; average correlations between all pairs of reproduction and longevity traits were positive (Additional file 2, Table S2). However, we found one exception to this trend for isolate-specific trait correlations: the three high-*ND5 *deletion *C. briggsae *isolates are significantly more likely to experience a tradeoff between early fecundity and longevity; *i.e.*, animals that live long tend to have reduced fecundity early in life (Figure 3) when such a reduction will most negatively affect population growth rates. These findings are reminiscent of those for *C. elegans *long lived mutants assayed either under nutritional stress [22] or benign conditions [24] and are consistent with the idea that the tradeoffs predicted by life history theory [25] may only manifest themselves in nematodes experiencing some form of stress - either exogenous or endogenous stress (*e.g.*, mitochondrial dysfunction).

**Figure 4:**
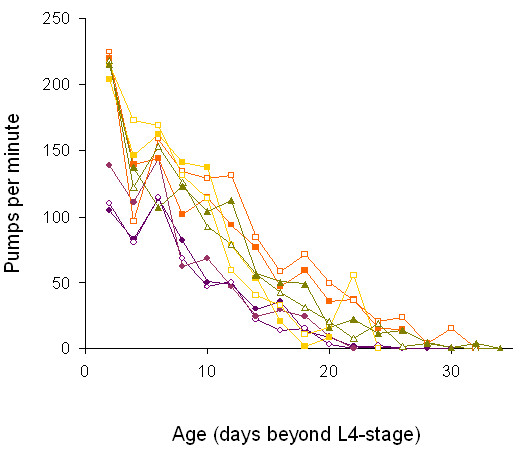
**Decline in *C. briggsae *isolate-specific pharyngeal pumping rates with age**. Each data point represents the mean of 3 replicate counts for each of group of animals. Pumping rates were measured for the same animals two days after the L4 stage and every two days until death. Isolates are color coded as in Figure 3 where zero deletion isolates = triangles, low deletion isolates = squares, and high deletion isolates = circles.

Because pharyngeal pumping rate is a reliable biomarker of age in *C. elegans *[26,27], we hypothesized that high-*ND5 *deletion *C. briggsae *isolates would exhibit slower pharyngeal pumping in non-aging individuals as well as faster declines in pharyngeal pumping (*i.e.*, increased muscle deterioration) with age when compared to low and zero-*ND5 *deletion isolates. In agreement with the first expectation, we observed a striking negative relationship between *ND5 *deletion level and average pharyngeal pumping rates in non-aging worms (Table 1, Figure 4). This trend persisted with increasing age; however, high deletion strains exhibited a somewhat slower rate of decline in pharyngeal pumping rates compared to low and zero-deletion isolates (Tukey HSD, α = 0.05, based on the difference in average pumping rate between days 2 and 20 post-L4; Figure 4). This may mean that high deletion isolates experience late-life reductions in their rates of aging compared to other isolates. Finally, we found significant natural variation in adult pharyngeal pumping rates throughout early but not late life (F-tests, p < 0.05 for time points prior to 14 days post-L4; analyses stopped after L4 + 20 days due to the reduction in statistical power caused by mortality). The magnitude of among-isolate differences in pumping rates was greatest during the young adult stage (Table 1; Figure 4) and declined slightly over the lifespan of the worms. Interestingly, the sharp decline in mean pumping rates at 4 days post-L4 coincided with the end of the reproductive period; this pattern is not observed in the congener, *C. elegans *[*e.g.*, [26], pers. obs.].

To further quantify natural phenotypic variation and to test whether the observed relationships between fitness-related life history traits and *ND5 *deletion levels may be mediated by mitochondrial ETC functioning, we quantified the relationship between *ND5 *deletion level and several aspects of animal physiology. Our expectation was that, as a result of reduced mitochondrial ETC efficiency, higher-*ND5 *deletion isolates would exhibit reduced levels of ATP and oxidative stress resistance, together with increased ROS production compared to lower or zero-*ND5 *deletion isolates.

**Figure 5:**
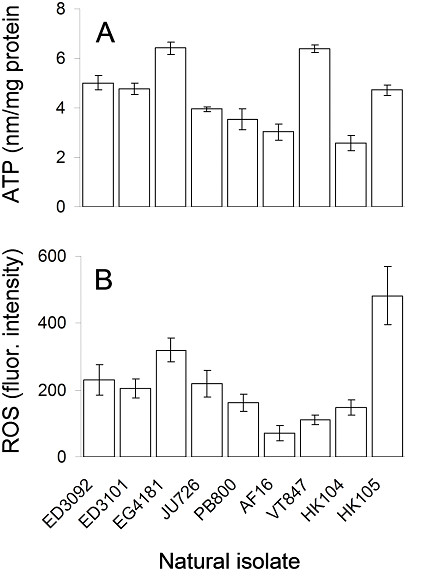
**Natural variation in ATP and ROS levels**. Natural variation in mean ATP content (A) and mean relative ROS levels in pharyngeal bulbs (B) among *C. briggsae *isolates. Isolates are arranged along the x-axis in order of increasing *ND5 *deletion level. Bars show one standard error. A second order quadratic provided the best fit to the relationship between relative superoxide level and *ND5 *deletion frequency (B), [adjusted R*** = 0.191, coefficient (SE) = 0.451 (0.093), p < 0.0001] even if the HK105 isolate was omitted from the analysis [adjusted R*** = 0.250, coefficient (SE) = 0.256 (0.064), p < 0.0001].

Only some of the above expectations were borne out by the data. We found significant among-isolate variation in average ATP content of young adult animals; however, there was only a weak, nonsignificant negative relationship between ATP content and *ND5 *deletion frequency (Table 1, Figure 5A). If high-*ND5 *deletion isolate, VT847 (Hawaii), which produced 6.379 ± 0.164 nM ATP/mg protein, was removed from the analysis, the correlation became more strongly negative (Pearson r = -0.370, p = 0.001). ATP production is known from other studies to sometimes be robust to ETC dysfunction; however, these data highlight the need to understand the degree to which nematodes may rely on alternate energy production pathways [28-30], which could obfuscate any pattern between ATP level and *ND5 *deletion frequency, for example.

We found significant among-isolate variation for oxidative stress resistance as measured by survival times of worms during acute paraquat exposure (Table 1). We predicted that, if higher-*ND5 *deletion isolates experience mitochondrial dysfunction leading to increased endogenous ROS levels, these isolates would be less able to survive attack from exogenous sources of ROS. A weak but significant negative relationship existed between this form of oxidative stress resistance and *ND5 *deletion frequency (Table 1). This pattern was largely driven by two low-*ND5 *deletion isolates (PB800 and JU726) with high oxidative stress resistance (data not shown). We currently have no information on whether *C. briggsae *isolates vary in their expression of antioxidant proteins and thus in their abilities to detoxify ROS, which would provide one possible explanation for the weak relationship between paraquat-induced oxidative stress resistance and *ND5 *deletion heteroplasmy level.

Finally, relative ROS levels measured for pharyngeal bulbs also varied significantly among *C. briggsae *isolates (Table 1; Figure 5B). We measured total levels of mitochondrial oxidants using MitoSOX Red (Invitrogen). MitoSOX Red is preferentially taken up by actively respiring mitochondria, and while it is nearly exclusively oxidized by superoxide [31,32]--the most common form of mitochondrially generated ROS--it is considered an accurate and effective system for measuring total ROS levels *in vivo *[32]. In contrast to our simple expectation that a positive, linear relationship would exist between *ND5 *deletion frequency and ROS level, we found a significant, non-linear relationship between these variables such that zero and high deletion isolates tended to exhibit higher superoxide levels than low deletion isolates (Figure 5B legend). The *in vivo *fluorogenic dye-based method used here gives a view of net ROS level in living tissue and therefore reflects both the rate at which ROS are generated by cells and the rate at which they are scavenged by superoxide dismutases and other antioxidant systems. Consequently, we do not yet know which of these sources of variability explain more of the variance in net ROS levels among isolates, which was considerable despite modest sample sizes (Table 1). This variation suggests that *C. briggsae *may provide a useful system in which to test whether endogenous ROS have a role in generating variability in spontaneous mutation rates [33].

Thus, while more work is required to understand whether and how the *ND5 *deletion directly affects mitochondrial functioning in *C. briggsae*, it is clear that there is no simple relationship between isolate-specific net ROS production and the detrimental phenotypes associated with high *ND5 *deletion heteroplasmy levels in nature. For example, both Japanese isolates, HK104 and HK105, exhibit low fitness (Figure 2) and pharyngeal pumping rates (Figure 4), but their relative levels of superoxide (and ATP) are quite different from one another (Figure 5). These results may indicate that *C. briggsae *isolates experience different forms of mitochondrial dysfunction [Figure 3 in [34]] that may or may not be related directly to the *ND5 *deletion.

The limited molecular population genetic structuring of *C. briggsae *[[35], but see [5]] belies the striking phenotypic variation among isolates reported here, the latter of which suggests an immense capacity for evolution of aging and life-history phenotypes. Together with previous findings of geographic variation in mitochondrial genome content among *C. briggsae *isolates [10] and of elevated spontaneous mutation rates in some isolates relative to the *C. elegans *N2 lab strain [6,19,20], the findings from this paper suggest that *C. briggsae *will provide an opportunity to understand the linkages between mitochondrial function (e.g., ROS production), mutation processes, and aging. And importantly, because *C. briggsae *contains *naturally-occurring *mtDNA variation that is likely to affect organismal fitness, this system offers a major advantage for studying the evolutionary dynamics of mtDNA mutations over *C. elegans *where the majority of mtDNA mutant strains have been isolated from laboratory mutagenesis screens.

## Conclusions

Although we found no straightforward relationship between *ND5 *deletion frequency and relative ROS levels, our accumulated findings are consistent with the notion that the natural mitochondrial genetic variation observed among *C. briggsae *isolates is detrimental to the organism. This is particularly true in light of recent studies that show high mutation rates in high-*ND5 C. briggsae *isolates [6,19,20], Nevertheless, two issues prevent us from demonstrating that the *ND5 *deletion is the root cause of the detrimental phenotypes: 1) *ND5 *deletion level is clearly confounded with phylogenetic relationship (*e.g., *two of our three "high-deletion" isolates are sister taxa from Japan; Figure 1), and 2) we cannot disentangle the effects of among-isolate variation in *ND5 *deletion level from among-isolate nuclear variation on the phenotypes studied here. However, the ability to generate inbred lines and mitochondrial-nuclear hybrid strains in which mitochondria from natural *C. briggsae *isolates with different *ND5 *deletion heteroplasmy levels may be evaluated on uniform nuclear genetic backgrounds [36] will be a major advantage of this nematode system. (A large-scale analysis of such hybrid strains will be reported elsewhere.) A congener of the well-known model nematode, *C. elegans*, *C. briggsae *has a similar life-history and mating system, and offers many of the same advantages as *C. elegans *as an animal genetic model system [37]. Together with the ample among-isolate variation in *ND5 *deletion heteroplasmy level (Figure 1B) [10] and aging-related phenotypes, we contend that the *C. briggsae *system offers a powerful and versatile model for understanding the interplay between mitochondrial dysfunction and aging in an evolutionary context, as well as the basic biology underlying mtDNA deletion genetics. This study lays the necessary groundwork to rigorously test these and other questions in aging biology and life history theory [*e.g.*, [38]].

## Methods

### Nematode strains and culture conditions

As described in [10], the *ΨND5-2 *pseudogene directly upstream of *ND5 *(Figure 1A) is required for the *ND5 *deletions to occur within *C. briggsae*. Directly repeated nucleotide sequences occur within *ND5 *and *ΨND5-2 *that promote direct repeat-associated deletion events. Isolates in the "Kenya" clade (Figure 1B) provide natural outgroup controls because they lack *ΨND5-2 *and are thus inherently unable to experience the *ND5 *deletions. The among-isolate variability in average heteroplasmy level is thought to also be partly accounted for by the presence of compensatory sequences within the mtDNA of certain populations (Figure 1B legend). These sequences, present at the *ΨND5-2 *direct repeat associated with the deletions, appear to prevent the deletion by reducing sequence homology between *ΨND5-2 *and *ND5 *and to thereby place an upper limit on the proportion of *ND5*-deletion bearing genomes able to accumulate within individuals [10]. We chose to use a subset of nine natural *C. briggsae *isolates studied by [10] that captured the full range of *ND5 *deletion heteroplasmy level (Figure 1, Additional file 1, Table S1). All nematodes were grown under standard laboratory conditions on 15 mm plates with NGM and *Escherichia coli *strain HB101. All nine natural isolates were included in each analysis.

### Life-history assays

Isolate-specific fecundities were estimated for each natural isolate following [39]. ''Early fecundity'' is the number of offspring produced on the first two days of reproduction combined; ''late fecundity'' is the number of offspring produced on the third and fourth days of reproduction. We also calculated total lifespan as the number of days lived from the egg stage. We calculated intrinsic rate of increase, *r*, for each natural isolate by solving Σe*** *l(x) m(x) *for *r*, where *l(x) *is the proportion of worms surviving to day *x *and *m(x) *is the fecundity on day *x*. We also quantified average correlations among life history traits across *C. briggsae *isolates to investigate whether the magnitude or pattern of these trait associations would change with increasing *ND5 *deletion frequency.

### Pharyngeal pumping

We measured the decline of pharyngeal pumping rate with age among *C. briggsae *isolates. Caenorhabditid nematodes ingest soil bacteria using rhythmic contractions of the pharynx - a neuromuscular organ comprising 20 muscle cells and 20 neurons. As in humans, muscle organization and function in nematodes deteriorates with chronological age. The rate of pharyngeal pumping is known to decline steadily with age in *C. elegans *as a result of contraction-related cellular injury [27,40]. Pharyngeal pumping was quantified for the same 10 to 20 individuals per isolate beginning two days after the L4 stage, and every other day until death. Daily pharyngeal pumping rates were taken as the average of three 5-second counts, converted to pumps per minute.

### ATP content

ATP content was measured using a protocol adapted from [[41], B. Braeckman, pers. comm.] for 8-10 independent, age-synchronous samples of young adult nematodes from each isolate. ATP content was determined for 50 uL of supernatant using the ATP Bioluminescence Assay Kit CLS II (Roche) and following the manufacturer's instructions. ATP measurements were standardized by total protein content using a BCA Kit (Pierce).

### Oxidative stress resistance

We measured resistance of *C. briggsae *to acute paraquat exposure following [42]. Age-synchronized populations of post-reproductive (8 day old) worms were generated for each natural isolate. Sixteen individuals per isolate were exposed to 300 mM paraquat in S-basal and scored for survival every 5 minutes. Worms that remained completely unresponsive after three taps with a glass prod were scored as dead.

### Relative superoxide production

We followed the basic approach of [31] to quantify relative ROS levels among *C. briggsae *isolates. Briefly, age-synchronized worms were incubated for 24 hours in 10 uM MitoSOX Red (Invitrogen) before being transferred as young adults to fresh NGM plates seeded with non-labeled HB101 *E. coli*. They were allowed to feed for 1 hour, and paralyzed using a drop of 5M levamisole prior to imaging. Images were acquired using a high resolution wide field Core DV system (Applied Precision™), equipped with an Olympus IX71 inverted microscope mounted with a Nikon Coolsnap ES2 HQ camera (Advanced Light Microscopy Core Facility, Oregon Health and Science University, Portland, OR). Fluorescent, z-stack images of the mitochondrial-rich pharynx with a 1.0 second exposure time were captured at 60X magnification. Images were deconvolved prior to analysis. Terminal pharyngeal bulbs were manually circled to quantify mean fluorescence intensity of the area in exposed and unexposed (control) animals for each strain using ImageJ software (NIH). The final pharyngeal bulb intensity values were calculated as the difference between intensity values for exposed and control worms.

Although the use of mitochondria-specific dyes has become a widely used and accepted method for measuring a variety of mitochondrial traits *in vivo*, a caveat is that differential dye uptake between samples could lead to inaccurate interpretation of fluorescence differences. There is currently no means of determining the extent to which this occurs [31]. However, great care was taken to minimize all other sources of error. For example, we exposed only one sample at a time to the microscope light source and used the shortest exposure time possible to avoid introducing variation due to breakdown of the dye.

### Statistical analyses

We analyzed among-isolate variation using separate one-way analyses of variance (ANOVA) for each phenotype measured. To test for differences between pairs of isolates, least-squares contrasts (Tukey's HSD for all pairwise comparisons; [43]) were performed on the data for each trait. To test for associations between traits and *ND5 *deletion levels, we calculated Pearson correlation coefficients between trait values and isolate-specific *ND5 *deletion percentages, and applied one-way ANOVA to data categorized as being from high, low, or zero-*ND5 *deletion isolates (see Figure 1B legend). Finally, we characterized correlations among life-history characters measured on the same individuals by calculating Spearman rank correlation coefficients between each pair of traits following [26].

## Authors' contributions

ALC-H and KAH carried out the metabolic and oxidative stress assays. KAH, SRM, JBK, and SWS carried out the life-history assays. KAH, KCS, and GdH carried out the reactive oxygen species assays. SE oversaw all experiments and data analysis, and drafted the manuscript, and DRD provided nematode stocks and helped draft the manuscript. All authors read and approved the final manuscript.

## Supplementary Material

Additional file 1 **Table S1**. *ND5 *deletion heteroplasmy data. Click here for file

Additional file 2 **Table S2**. Life-history trait correlations. Click here for file
