# Supplementary material for: Natural variation in life history and aging phenotypes is associated with mitochondrial DNA deletion frequency in Caenorhabditis briggsae
Source: BMC Evol Biol. 2011 Jan 12;11:11. doi: 10.1186/1471-2148-11-11 (PMC3032685; doi:10.1186/1471-2148-11-11)
Supplement: Additional file 1 — Table S1. ND5 deletion heteroplasmy data. [file 1471-2148-11-11-S1.DOC]

**Supplementary Table 1. Life-history trait correlations.**

|  | Late | Total | *R* | Lifespan |
| --- | --- | --- | --- | --- |
| Early fecundity | 0.247^***^ | 0.487^***^ | 0.693^**^ | 0.047 |
| Late fecundity |  | 0.950^***^ | 0.688^**^ | 0.162^*^ |
| Total fecundity |  |  | 0.818^**^ | 0.168^*^ |
| *R* |  |  |  | 0.143 |

Spearman's correlation coefficients for each pair of life history traits in *C. briggsae* isolates. ^*^, ^**^, and ^***^ denote significant differences from 0 at the 0.05, 0.01, and 0.001 levels, respectively.
