# Supplementary material for: Natural variation in life history and aging phenotypes is associated with mitochondrial DNA deletion frequency in Caenorhabditis briggsae
Source: BMC Evol Biol. 2011 Jan 12;11:11. doi: 10.1186/1471-2148-11-11 (PMC3032685; doi:10.1186/1471-2148-11-11)
Supplement: Additional file 2 — Table S2. Life-history trait correlations. [file 1471-2148-11-11-S2.DOC]

**Supplementary Table 2. *ND5* deletion heteroplasmy data.**

|  | **Natural Isolate (in order of increasing ND5%)** | | | | | | | | |
| --- | --- | --- | --- | --- | --- | --- | --- | --- | --- |
| **Banding Pattern** | ED3092 | ED3101 | EG4181 | JU726 | PB800 | AF16 | VT847 | HK104 | HK105 |
| Intact only | 8 | 8 | 0 | 0 | 0 | 0 | 0 | 0 | 0 |
| Large only | 0 | 0 | 6 | 4 | 2 | 0 | 0 | 0 | 0 |
| Large+small | 0 | 0 | 2 | 4 | 6 | 5 | 1 | 0 | 0 |
| Small only | 0 | 0 | 0 | 0 | 0 | 3 | 7 | 8 | 8 |
| ***ND5*%** | 0 | 0 | 11 | 18 | 24 | 40 | 56 | 60 | 60 |

Howe and Denver [10] quantified *ND5* deletion heteroplasmy levels using qPCR and conventional PCR-based analyses of L1-stage nematodes (Fig. 1). To verify that average *ND5* deletion heteroplasmy levels were the same in young adult-stage animals, we applied the latter of these approaches to DNA extractions from single young adult nematodes from each natural isolate. This qualitative assay used primers flanking the *ND5* deletion area and produced banding patterns: Intact only = intact genomes lacking the pseudogenetic element, *ΨND5-2*, only observed; Large only = intact genomes containing *ΨND5-2* only observed; Large+small = intact and deletion-bearing genomes containing *ΨND5-2* observed; Small only = deletion-bearing genomes only observed. The assay produced only single large amplicons in nematodes where ND5 deletion-bearing genomes are ~5% of the total, both a large and a small amplicon in nematodes where deletion levels are ~30%, and only the small amplicon in cases where deletion levels are ~60% [see 10]. Using these rough guidelines, we estimated *ND5* deletion heteroplasmy levels (***ND5*%**) among young adult animals to be slightly higher than previous estimates from L1-stage animals, but of an identical pattern in terms of the rank order of isolate-specific heteroplasmy levels (Fig. 1). We used the more quantitative estimates of *ND5*% in Fig. 1 for all analyses.
